# Blast Resistance Capacities of Structural Panels Subjected to Shock-Tube Testing with ANFO Explosive

**DOI:** 10.3390/ma16155274

**Published:** 2023-07-27

**Authors:** Gang-Kyu Park, Jae Heum Moon, Hyun-Seop Shin, Sung-Wook Kim

**Affiliations:** Department of Structural Engineering Research, Korea Institute of Civil Engineering and Building Technology, 283 Goyangdae-ro, Ilsanseo-gu, Goyang-si 10223, Gyeonggi-do, Republic of Korea; mjh4190@kict.re.kr (J.H.M.); hsshin@kict.re.kr (H.-S.S.); swkim@kict.re.kr (S.-W.K.)

**Keywords:** HPFRCC, polyurea, energy-absorbing panel, shock-tube test

## Abstract

This study presents a series of shock-tube tests conducted on structural panels using ammonium nitrate fuel oil (ANFO) as the explosive. The characteristics of the blast waves propagating through the shock tube were analyzed by measuring the pressure generated at specific locations inside the shock tube. The extent of differences in blast pressure generated in a confined space, such as the shock tube, was compared to that predicted by the proposed method in the Unified Facilities Criteria 3-340-02 report. The target specimens of this study were plain reinforced concrete (RC), high-performance fiber-reinforced cementitious composites (HPFRCCs), and composite panels. Polyurea-coated RC panels and steel plate grid structure-attached RC panels were used as composite panels to evaluate the effectiveness of the coating and structural damping methods on the enhancement of structural blast resistance. The tests were conducted with different ANFO charges, and the crack patterns and lengths on the rear surface of each panel were measured. Based on the measured results, discussions regarding the blast resistance capacities of each panel type are provided.

## 1. Introduction

With increasing concerns regarding explosion incidents, such as bomb attacks, improvement of the blast resistance of concrete structures has become an important factor to be considered in structural design. When concrete structures are subjected to extreme loads of high amplitudes and short durations, considerable amounts of debris and fragments are generated on the rear face of the concrete structures owing to the brittle characteristics of concrete. Because this phenomenon can result in secondary damage to facilities and human lives inside protective structures, various reinforcement methods have been developed to compensate for the inherent drawbacks of concrete. To improve material performance, different fibers have been introduced in various cement-based composite materials that have high energy absorption and ductility properties; some of them are ultrahigh-performance concrete (UHPC) and high-performance fiber-reinforced cement composites (HPFRCCs) [1,2,3,4]. Furthermore, various materials, such as fiber-reinforced polymer (FRP) [5,6] and polyurethane [7,8], have been utilized to enhance the tensile performance and minimize the structural debris, thereby improving structural performance. Energy-absorbing panels [9,10] have been developed to absorb the energy generated by explosions.

Many studies have investigated the blast-resistance performance of structures using improved cement-based materials. Li et al. [11] manufactured three reinforced concrete 10 (RC) slabs and three ultrahigh-toughness cementitious composite (UHTCC) slabs using PVA fibers to compare the resistance capacity under contact explosion using TNT charges. Severe spalling damage was observed on the bottom faces of the RC slabs, whereas reduced spalling damage or no damage was identified in the UHTCC slabs having excellent energy absorption ability. Xu et al. [12] compared the damage characteristics and failure modes of reactive powder concrete (RPC) and high-strength concrete (HSC) targets based on different explosive embedment depths at the center of the targets. They compared the crater area and depth of the RPC and HSC targets, and the results revealed that the RPC specimens demonstrated better resistance and integrity, and they generated less debris against the explosion. Additionally, the damage was observed to be more severe when the depth of the explosive charge was closer to the center of the target. Kim et al. [13] produced high-performance fiber-reinforced cementitious composite (HPFRCC) specimens with long steel and nylon fibers and tested them under contact explosion and military landmine detonation conditions. The maximum damage depth and mass-change rate were measured after the experiment. It was found that using only nylon fibers did not provide adequate resistance against explosion, and the specimens reinforced with both long steel and nylon fibers demonstrated better explosion resistance performance compared to those reinforced with only long steel fibers.

In addition to the development of cement-based materials to improve material properties, many studies have focused on enhancing structural performance by applying various reinforcing materials to the interior or exterior of structures. Ha et al. [14] conducted a free-air burst test with 15.88 kg ammonium nitrate fuel oil (ANFO) explosive at a standoff distance of 1.5 m to investigate the failure characteristics of plain RC panels retrofitted with either carbon fiber-reinforced polymer (CFRP), polyurea (PU), or a combination of CFRP and PU. The retrofitted specimens exhibited superior performance in terms of displacement, energy absorption, and spalling compared to the plain RC specimen. Among the retrofitting materials, CFRP and PU provided the best blast resistance. Xu et al. [9] performed contact explosion tests on a normal-strength concrete slab and three geopolymer-based UHPC (G-UHPC) slabs embedded with a steel wire mesh (SWM) and energy-absorbing foam materials. Among the different combinations, G-UHPC slabs embedded with both aluminum honeycomb panels (AHPs) and polyurethane foam panels (PFPs) exhibited the best performance against contact explosion. It was recommended to consider using energy-absorbing foam materials to improve the blast resistance of concrete slabs. An experimental study on the failure modes and dynamic responses of RC slabs and steel–concrete (SC) slabs under contact explosion was conducted by Zhao et al. [10]. It was reported that although single-sided steel–concrete (SSSC) and center SC slabs exhibited greater crater areas than RC slabs, the slabs retained their integrity without penetration and exhibited reduced mid-span deflection owing to the existence of the steel plate.

Several experimental studies have been conducted to evaluate the resistance performance of structures subjected to blast loads; however, their results were mostly obtained from field tests resulting in large variations and a lack of reproducibility [15]. Therefore, in this study, explosion tests were performed using a shock tube to form reproducible shock waves and ensure the reliability of the experimental results. Powder-type ANFO was used as the explosive, and the characteristics of the resulting incident waves passing through the shock tube were analyzed according to the measurement points. Using normal-strength reinforced concrete panels as control specimens, the HPFRCC, polyurea, and energy-absorbing panels were considered as variables in the experiment. The failure modes and crack lengths were investigated according to the amount of ANFO explosive to compare the resistance performances of the structures.

## 2. Experimental Program

### 2.1. Sample Preparation

The mix proportions of normal-strength concrete and HPFRCC are listed in Table 1. For normal-strength concrete, type Ⅰ Portland cement, ground granulated blast furnace slag (GGBS), fine aggregates, and coarse aggregates were used. The maximum sizes of the fine and coarse aggregates were 5 and 25 mm, respectively. Type Ⅰ Portland cement, silica fume (SF), and fly ash (FA) were used as the HPFRCC matrix, and coarse aggregates were excluded to render the internal structure of the cementitious matrix denser. Domestic sand No. 6 with an average size of approximately 294 μm was adopted for a design matrix strength of 100 MPa. The average sizes of SF and silica flour were 0.31 and 14.1 μm, respectively. A polycarboxylate superplasticizer (SP) with a density of 1.01 g/cm^3^ was introduced into the cementitious matrix to compensate for the insufficient flowability owing to the low water-to-binder ratio (W/B). A glycol-based shrinkage-reducing admixture (SRA) was used to mitigate the potential for shrinkage cracks during production. Straight steel fibers with a 2% volume fraction were added to the HPFRCC mixtures, and the aspect ratio ($l_{f}/d_{f}$) of the steel fibers was 97.5, where $d_{f}$ and $l_{f}$ are the diameter and length of the fiber, respectively. The material properties of the steel fibers used in this study are summarized in Table 2.

**Figure 1:**
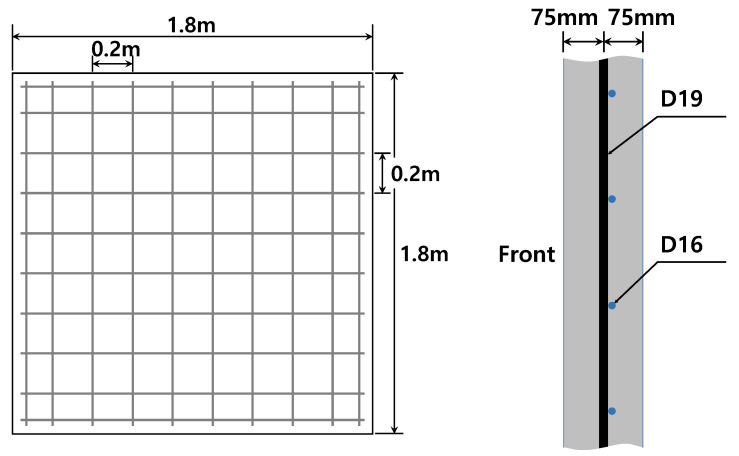
Representation of the panels for the shock-tube test.

Table 3 summarizes the details of the panels used in the shock-tube experiments. The specimens were classified according to their matrix strengths, thicknesses, and reinforcement materials. Figure 1 presents the panel specimens for the shock-tube tests with a size of 1.8 m × 1.8 m × T, where T is either 150 mm or 100 mm. SD 400 reinforcing steels with diameters of 19 and 16 mm were used; the reinforcing bars were spaced at 200 mm intervals, both vertically and horizontally. For the normal-strength concrete panels, 12 specimens were cast on the same day using ready-mixed concrete and subjected to air-curing conditions. For the HPFRCC panels, six specimens were cast in molds after mixing using a Hobart-type mixer. The specimens were initially cured for a day at room temperature, and then, they were steam-cured in a chamber at a high temperature of 90 °C for 3 d. The steam-cured panels were cured at room temperature until tested. As the panels were manufactured, additional cylindrical samples of a height and diameter of 200 and 100 mm, respectively, were cast simultaneously and tested according to KS F 2405 [16], which is similar to ASTM C 39 [17], on the first day of the shock-tube test. The average compressive strength of normal-strength concrete and HPFRCC were 32.6 MPa and 112 MPa, respectively.

**Figure 2:**
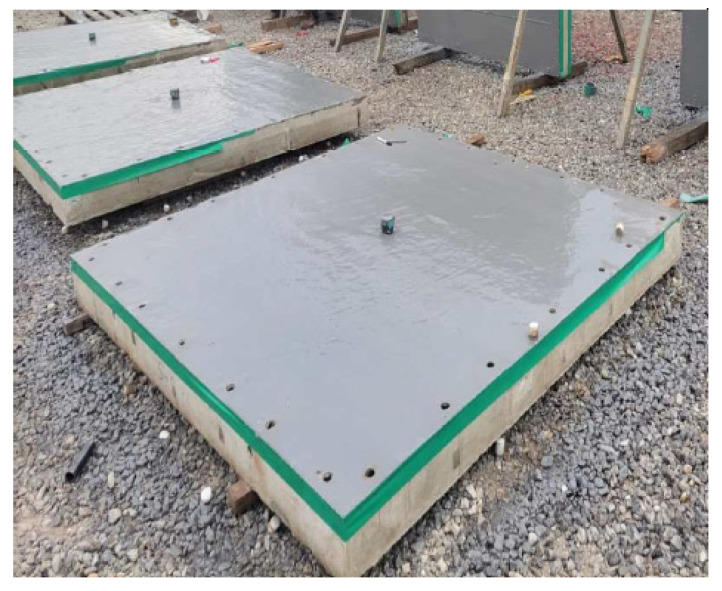
Polyurea-coated specimens.

**Figure 3:**
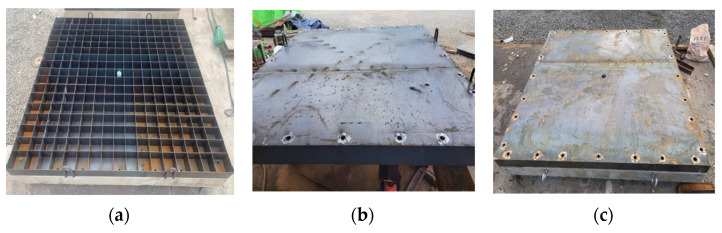
Fabrication of energy-absorbing panels: (**a**) Steel grid; (**b**) Welding front/back steel plates; (**c**) Test specimen.

In this study, polyurea and energy-absorbing panels were used as the variables for structural reinforcement. As shown in Figure 2, polyurea was applied to the rear faces of the specimens based on the manufactured normal-strength concrete panels. The tensile strength and elongation of the polyurea were 16 MPa and 450 ± 100 (%), respectively. The thickness of the polyurea was determined as the average of the thickness measured at eight points on four sides of the specimens. The total thicknesses of the three polyurea-coated panels were 154.10 mm, 153.78 mm, and 153.53 mm, respectively. As shown in Figure 3, the energy-absorbing panels were fabricated in the form of a grid to absorb energy when the grid-type panel was distorted. Two grid dimensions were considered: 100 mm × 100 mm and 50 mm × 50 mm. The grid type-panel comprised thin steel plates of a height and thickness of 100 and 2 mm, respectively (see Figure 3a). Additionally, a thin steel plate of a thickness of 2 mm was attached to the front and back of the grid-type panel (see Figure 3b). SPHC steel with a tensile strength of 350 MPa was used for fabricating the panels. The manufactured energy-absorbing panel was placed on the front face of the normal-strength concrete panel.

### 2.2. Shock-Tube Test

**Figure 4:**
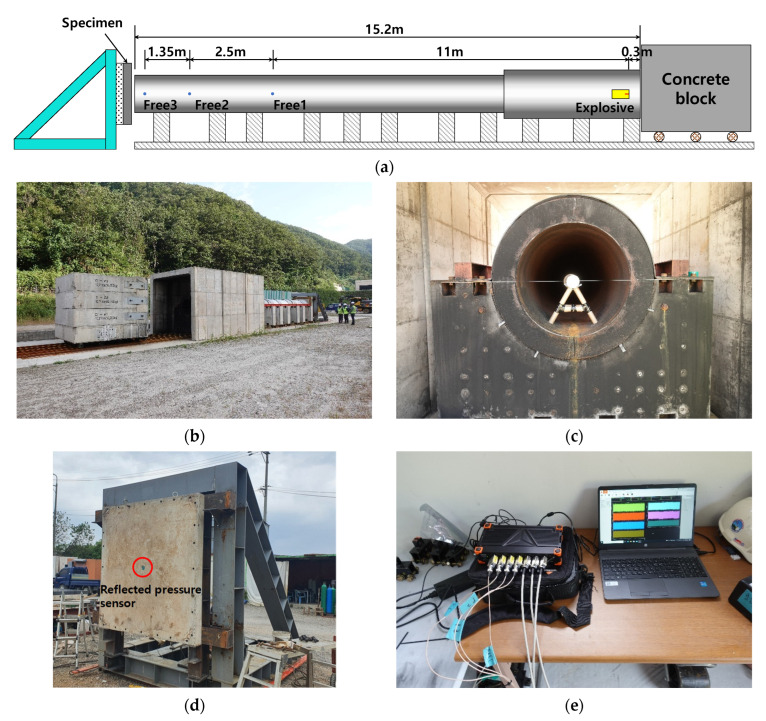
Experimental setup of the shock-tube test: (**a**) Schematic of shock-tube test setup; (**b**) Actual shock tube test setup; (**c**) Placement of the ANFO explosive; (**d**) Specimen installation; (**e**) Data logger.

Figure 4a shows the shock-tube apparatus used in this study. The inner diameter and length of the tube were 1 and 15.2 m, respectively. This was designed for the initiated blast pressure to travel sufficiently through the tube before the shock pressure was applied to the test specimens. Compared with open field explosion tests, the shock-tube test minimizes the effects of environmental conditions on the test results and enhances the reliability of the test results by applying controlled and consistent pressures. Powder-type ANFO was used as the explosive. ANFO charges of 1, 2, and 3 kg were considered for all specimens in this study. The charge amount was determined through several preliminary tests to prevent excessive deformation of the rigid frame located behind the specimen. ANFO powder was filled in the cylindrical paper container, and the container was placed at the inner center, 300 mm away from the back end of the shock tube (Figure 4a). An additional primer (booster) of 100 g was used to initiate the explosion. Prior to the test, the back end of the shock tube was closed using a heavy-mass concrete block with wheels on the rails, as shown in Figure 4b. The shock impact on the shock tube was reduced by allowing the movement of the concrete block to release a certain amount of pressure from the back end of the shock tube when an explosion occurs. The blast pressure from the front end of the shock tube was applied to a panel-type test specimen that was fixed to the rigid frame using multiple bolts. A distance of 100 mm between the front end of the shock tube and front surface of the test specimen was maintained in this study. For different specimen thicknesses, the rigid frame, which was fixed on the strongly reinforced concrete base, was relocated to maintain the distance between the test specimen and front end of the shock tube.

To measure the magnitude of the blast wave passing through the shock tube, three pressure gauges (HEL-375CO-500A, Kulite, Leonia, NJ, USA) were installed 11 m, 13.5 m, and 14.85 m away from the ANFO source to identify the superposition of blast load within the shock tube, as shown in Figure 4a. Figure 4d displays a photograph of the specimen mounted on the steel frame where a reflected pressure sensor (2300V4, Dytran Instruments Inc., Chatsworth, CA, USA) was attached to the steel pipe embedded at the center of the specimen. Through several preliminary tests, it has been observed that placing the sensor at the center of the specimen can be representative of applied load on the specimen. An accelerometer (M350D02, PCB Piezotronics Inc., Depew, NY, USA) was attached to the steel tube on the rear side of the specimen. According to the experimental program, the measured data from the sensors were obtained from the data logger (SIRIUSi-HS 8xSTG, DEWESOFT, Trbovlje, Slovenia) (see Figure 4e) with a sampling rate and duration of 500 kHz and 100 ms, respectively.

## 3. Experimental Results and Discussions

### 3.1. Characteristics of Incident Pressure in the Shock-Tube

**Figure 5:**
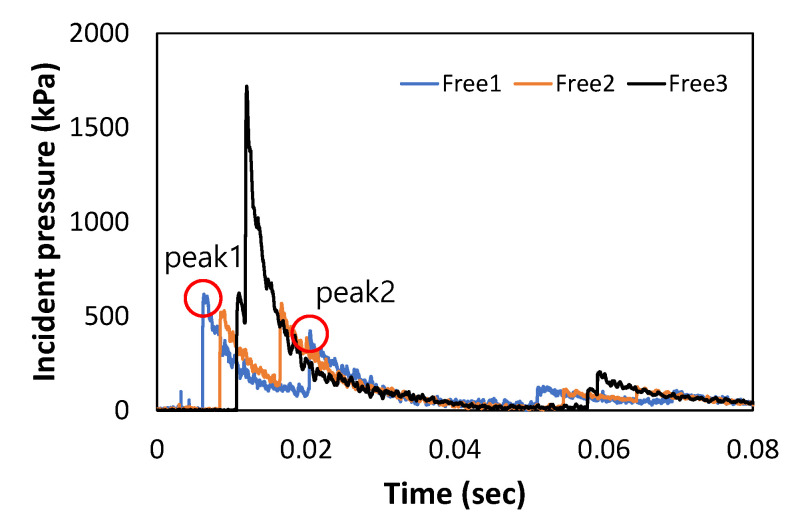
Representative results for the incident pressure–time curves based on measurement location (ANFO 2 kg).

Figure 5 presents the representative results for the incident pressures measured in the shock tube with 2 kg of ANFO. ‘Free 1’ refers to the incident pressure measured at the nearest location from the explosion source, and ‘Free 3’ refers to the incident pressure measured at the farthest location from the explosion source (see Figure 4a). Figure 5 confirms that there are several peaks for each pressure–time curve. This was because the generated pressure was reflected by the concrete block located at the back end of the shock tube, which resulted in one or two additional peaks after the first peak in the curves. In this study, the first two peak pressures were used to characterize the shock waves of the ANFO charge in the shock-tube test, and the third peak pressure was ignored because of its relatively small magnitude and late occurrence.

**Figure 6:**
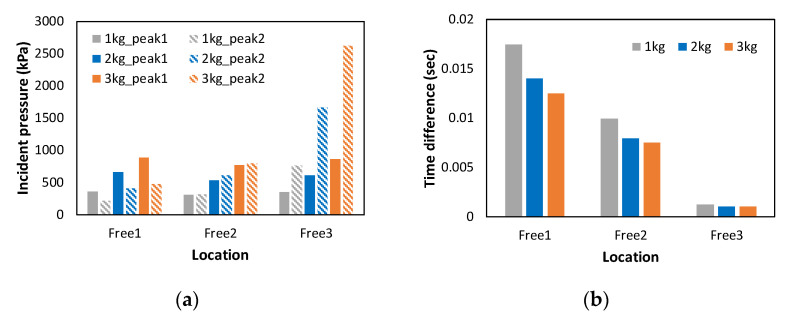
Averaged experimental results according to the amount of ANFO explosives: (**a**) Incident pressures at the measurement locations; (**b**) Time differences at the measurement locations.

Table 4 summarizes the first and second peak pressures and the time difference between the two peaks according to the measurement location and amount of ANFO charge. The average pressure and time differences are shown in Figure 6. From the table and figure, it is seen that the magnitude of pressure increases with an increase in the ANFO charge. In the case of Free 1, the first peak pressure was larger than the second peak pressure. For Free 2, no significant difference was observed between the first and second peak pressures. In contrast, at the Free 3 location, the second peak pressure was much higher than the first peak pressure. In addition, it was confirmed that the time difference between the first and second peak pressures decreased when the measurement point was further away from the explosion source. This implies that the second shock wave reflected by the concrete block passes through the shock tube faster because the first shock wave causes the air pressure in the shock tube to drop below the atmospheric pressure. There was no overlap between the first and second pressures owing to the large time difference at the Free 1 and Free 2 locations, whereas the pressure was amplified immediately after the first peak pressure owing to the overlap of the first and second pressures at the Free 3 location. Moreover, as shown in Figure 6b, the time difference between the first and second peaks gradually decreased as the ANFO charge increased for all measuring points, indicating that the velocity of the generated shock wave increased with an increase in the ANFO charge.

The measured pressures in the shock tube and the calculated pressures were obtained from the procedure proposed in the Unified Facilities Criteria (UFC) 3-340-02 report [18]. The incident pressure can be calculated from a given scaled distance, which is a function of the charge weight (*W*) and the ground distance (*R*). In this study, the TNT equivalent of ANFO was set to 0.74, incident pressures were calculated based on the graphs provided in [18], and free-air burst blast and surface burst blast conditions were considered. The pressures calculated from the report and the average pressures based on the results with the same ANFO charge weight in the experiment are summarized in Table 5. There was a significant difference between the results of the UFC report and those of the shock-tube tests. The graphs provided by the UFC report were obtained through numerous experiments in an open field, where no reflection or only ground reflection of the blast pressure was considered. However, the pressures measured at the same distance in the shock tube differed significantly from those in the UFC report because the generated pressure was continuously superimposed as it passed through the shock tube. Therefore, it can be concluded that the procedure proposed by the UFC report for the estimation of incident pressure is not applicable to the shock-tube test because of the different boundary conditions in the open field.

### 3.2. Explosion Resistance Performance of the Panels

**Figure 7:**
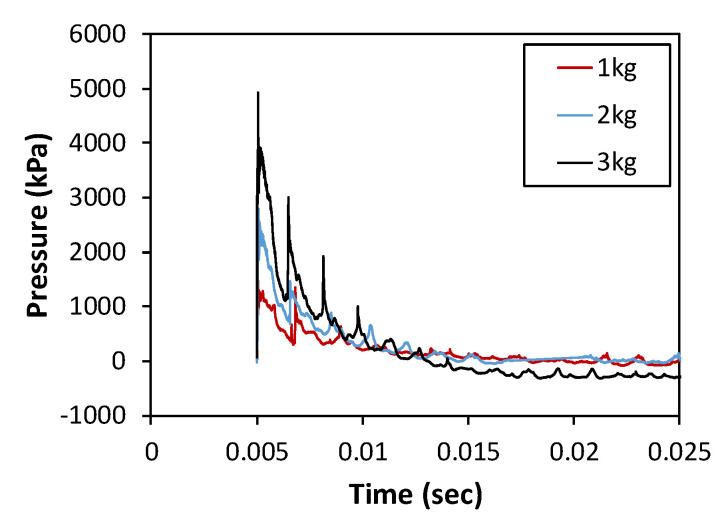
Representative results for the incident pressure–time curves based on measurement location.

The experimental results for each specimen obtained by performing the shock-tube test, including the acceleration, reflected pressure, duration, and crack length at the rear face, are summarized in Table 6. A representative result of the reflected pressure obtained from the N-150 specimen according to the amount of ANFO charge is presented in Figure 6. Similar to the results of the pressure measured in the shock tube, several peaks were observed, owing to the reflection of pressure by the concrete block located at the back end of the shock tube. From Figure 7 and Table 6, the reflected pressure and impulse gradually increased with increasing ANFO charge. The duration of the reflected pressure tended to decrease because of the faster velocity of the shock wave as the ANFO charge increased. For N-150-EAP-100, a low impulse value was measured. As shown in the experimental results of N-150-EAP-100 in Table 7, the welding zone on the front face of the energy-absorbing panel was separated owing to the low welding quality. The pressure flowed inside the panel instead of being applied to the front face of the specimen; thus, the reflected pressure could not be measured accurately.

**Figure 8:**
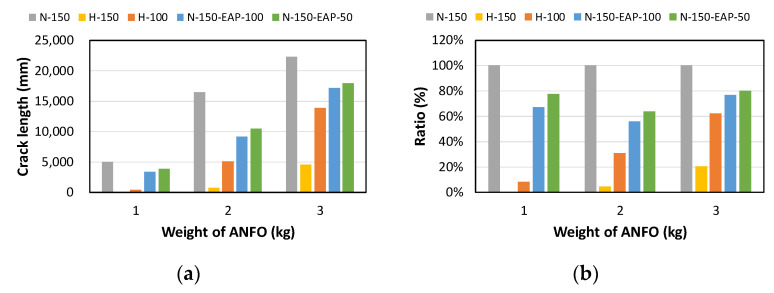
Comparison of crack lengths for the tested specimens according to the amount of ANFO explosives: (**a**) Measured crack length; (**b**) Reduction ratio of crack length.

The total crack lengths on the rear faces of the specimens were measured using an image-processing program after objectification by preprocessing all the images to the same pixel and scale. Based on the measured crack length, the resistance capacities of the specimens against the blast wave were compared, and the experimental results according to the ANFO charge weight are presented in Table 6 and Figure 8. Table 7 shows the experimental results for both the front and rear faces of all the specimens subjected to the shock-tube test with an ANFO charge of 3 kg. For the cases with other ANFO charges, crack damage on the front face and spalling or severe damage on the rear faces were not observed for any of the specimens. In the case of the polyurea-coated specimens, no tearing of the polyurea was observed, regardless of the amount of ANFO charge; thus, the crack lengths of these specimens were not measured.

As shown in Table 7, the control specimen N-150 was found to have incurred the most damage and a circle-shaped crack pattern on the front face, similar to the shape of the shock tube. A large fragment of concrete on both sides separated from the specimen along with a large crack width and length. The specimens using HPFRCC materials were found to have the best blast resistance performance among the specimens considered in this study, and even the thinner H-100 specimens exhibited better performance than the others. Unlike the N-150 specimen, the other two specimens exhibited no spalling on their rear faces and relatively few cracks. This can be attributed to the properties of the HPFRCC material, such as high compressive strength and improved energy absorption capacity induced by the bridging effect between cracks, which resulted in a significant reduction in crack propagation. For the polyurea-coated specimen, the damage to the front face of the specimen was reduced owing to the reinforcing effect of polyurea on the tensile behavior of the specimen. It was observed that no debonding occurred between the specimen and polyurea, and the use of a 3 kg ANFO explosive did not generate a sufficient blast load to exceed the tensile strength of the polyurea. Therefore, the application of polyurea seems to be an effective method of reinforcing the tensile resistance capacity of a specimen and preventing the scattering of fragments from the rear face. Compared to the control specimen of N-150, the specimens with energy-absorbing panels showed relatively fewer cracks on the rear face. However, the degree of reduction was insignificant. By comparing the crack lengths of the N-150-EAP-100 and N-150-EAP-50 specimens, it was confirmed that there was little difference in the structural performance between the two panels, except for the small spalling area on the rear face of the N-150-EAP-100 specimen. The aim of the energy-absorbing panel was to reduce the occurrence of cracks on the rear face of the specimen by absorbing the blast energy as the panel deformed, thereby minimizing the energy transferred to the concrete specimen behind the panel. However, the energy-absorbing panels did not show noticeable deformation owing to their high stiffness, indicating that most of the blast energy was applied to the concrete specimen behind the panel instead of being absorbed by the panel. Therefore, as shown in Figure 8b, the occurrence of cracks was reduced by approximately 20% compared with the control specimen N-150. In addition, the blast resistance of the specimens with energy-absorbing panels was much lower than that of the HPFRCC specimens. Therefore, for an effective energy absorption, it is necessary to reduce the stiffness of the energy-absorbing panel through widening the grid spacing or using steel with lower strength and stiffness.

## 4. Conclusions

This study investigated the characteristics of blast waves in a shock tube with an ANFO explosive and compared the blast resistance capacities of the considered specimens. By adopting plain reinforced concrete panel as the control specimen, the application of polyurea, an energy-absorbing panel, and HPFRCC were considered as variables. The following conclusions were drawn from the shock-tube tests:

1. Several peaks were observed in the pressure–time curve owing to reflection by the mass concrete block located at the back end of the shock tube. The time difference between the first and second peaks decreased with the distance from the explosion source, and the pressure was significantly amplified owing to the overlap of the first and second pressure peaks at the Free 3 location;
2. The blast pressure was amplified owing to multiple reflections of the pressure within the shock tube as the generated pressure passed through it. Thus, the measured pressure in the shock tube was significantly different from the pressure predicted by the method proposed in the UFC report;
3. Among the specimens considered in this study, the HPFRCC panels demonstrated the best performance against blast pressure even for the thinner specimen. This was attributed to the improved material properties and the inclusion of steel fiber to restrain crack propagation;
4. Polyurea can be effectively used to reinforce the tensile behavior of concrete structures and prevent the scattering of fragments;
5. Energy-absorbing panels used in this study were found to be ineffective with regard to their energy absorption capacity. To increase the energy absorption capacity, the stiffness of the energy-absorbing panels should be reduced to allow deformation of the panel to dissipate the blast energy.

## Figures and Tables

**Table 1:** Mix proportions of normal-strength concrete and HPFRCC.

		NC (*f_c_* = 30 MPa)	HPFRCC (*f_c_* = 100 MPa)
Water-to-Binder ratio		0.37	0.28
Mix Composition (kg/m^3^)	Water	122	248.4
	Cement	377	704.4
	Silica Fume	-	70.5
	Fly Ash	-	140.9
	Ground Granulated Blast Furnace Slag (GGBS)	42	-
	Silica Flour	-	140.9
	Silica Sand	-	845.3
	Fine Aggregate	831	-
	Coarse Aggregate	978	-
	Superplasticizer (SP)	-	11.1
	Shrinkage-Reducing Admixture (SRA)	-	4.6
	Steel Fiber (SF)	-	156.0

**Table 2:** Properties of steel fiber.

	Diameter (μm)	Length (mm)	Density (g/cm^3^)	Tensile Strength (MPa)	Elastic Modulus (GPa)
Steel fiber	200	19.5	7.8	2500	200

**Table 3:** Experimental variables for shock-tube test.

Specimen	Thickness (mm)	ANFO (kg)	Reinforcing Steel	Reinforcement	
				Variable	Remarks
N-150	150	1 kg 2 kg 3 kg	D19@200 mm (V) D16@200 mm (H)	-	-
H-150	150			HPFRCC	-
H-100	100				-
N-150-Po	150			Polyurea	-
N-150-EAP-100	150			Steel energy-absorbing panel	Grid size 100 mm × 100 mm
N-150-EAP-50	150				Grid size 50 mm × 50 mm

Note: N = normal strength concrete; H = HPFRCC; Po = polyurea; EAP = energy-absorbing panel.

**Table 4:** Obtained pressure and time difference according to measurement location and ANFO charge.

Specimen	ANFO (kg)	Free 1		Free 2		Free 3		Time Difference		
		Peak1 (kPa)	Peak2 (kPa)	Peak1 (kPa)	Peak2 (kPa)	Peak1 (kPa)	Peak2 (kPa)	Free 1 (s)	Free 2 (s)	Free 3 (s)
N-150	1	386.0	226.3	327.0	385.0	290.5	885.2	0.016644	0.00961	0.001268
	2	616.4	422.0	528.3	567.7	622.3	1719.2	0.014246	0.00768	0.001024
	3	888.9	517.6	771.4	820.1	1006.9	2849.5	0.012574	0.007276	0.00103
H-150	1	323.5	130.8	260.4	226.3	296.2	630.9	0.016886	0.01027	0.001142
	2	625.0	368.2	502.3	598.1	598.9	1590.6	0.01386	0.007988	0.001084
	3	726.8	310.0	636.4	657.8	703.2	2041.1	0.013594	0.008118	0.001048
H-100	1	342.3	147.5	280.8	237.6	311.8	674.4	0.016826	0.009602	0.00123
	2	840.4	413.2	541.5	631.9	608.4	1649.5	0.014286	0.007818	0.001096
	3	972.5	503.2	819.9	825.8	874.4	2548.3	0.012446	0.007236	0.000758
N-150-Po	1	323.0	172.0	287.0	301.4	312.9	713.0	0.016694	0.009464	0.00124
	2	622.6	360.5	525.6	682.4	597.9	1721.5	0.013596	0.00759	0.001058
	3	902.6	551.8	780.4	904.9	851.5	2881.4	0.012148	0.007312	0.001068
N-150-EAP-100	1	401.0	223.4	353.9	378.6	386.5	867.5	0.017044	0.009962	0.001212
	2	649.9	437.7	568.0	606.6	623.2	1696.8	0.013836	0.007896	0.000968
	3	913.2	462.5	824.6	741.6	879.0	2661.8	0.01181	0.007482	0.001156
N-150-EAP-50	1	388.7	235.4	351.6	340.3	381.0	867.0	0.016784	0.009962	0.001216
	2	620.6	463.7	524.2	581.9	593.3	1604.6	0.014282	0.008474	0.000968
	3	888.1	507.6	776.2	793.5	860.9	2739.3	0.012248	0.007574	0.001068

**Table 5:** Comparison of measured pressure with the calculated pressures from UFC report.

Location	ANFO (kg)	Scaled Distance (ft/lb^1/3^)	UFC 3-340-02 [18]		Experiment	
			Peak Pressure (Free ^a^) (kPa)	Peak Pressure (Surface ^b^) (kPa)	1st Peak Pressure (Peak1) (kPa)	2nd Peak Pressure (Peak2) (kPa)
Free 1	1 kg	30.66	8.96	12.41	360.76	211.98
	2 kg	24.33	13.10	15.86	662.49	410.89
	3 kg	21.26	14.13	20.00	882.02	475.42
Free 2	1 kg	37.62	6.34	10.00	310.11	311.54
	2 kg	29.86	9.65	12.41	531.66	611.43
	3 kg	26.09	12.07	14.13	768.13	790.61
Free 3	1 kg	41.39	5.86	7.58	346.50	944.29
	2 kg	32.85	8.62	11.03	607.34	1663.68
	3 kg	28.70	10.34	12.41	862.67	2620.23

Note: ^a^ refers to the free-air burst blast environment, ^b^ refers to the surface burst blast environment.

**Table 6:** Experimental results of the shock-tube test.

Specimen	ANFO (kg)	Acceleration (g)	Reflected Pressure (kPa)	Impulse (kPa∙s)	Duration (s)	Crack Length (mm)
N-150	1	2983.37	1750	3.72	0.01563	5028.1
	2	3221.26	2792	5.24	0.01202	16,485.5
	3	-	4909	7.50	0.00810	22,357.8
H-150	1	2243.77	1389	3.72	0.01129	-
	2	3268.71	2806	5.24	0.01123	804.9
	3	5800.75	4098	7.50	0.01039	4601.8
H-100	1	1498.88	1480	2.94	0.01119	427.1
	2	3721.99	3743	6.15	0.01048	5130.9
	3	3366.87	4901	8.29	0.01004	13,911.9
N-150-Po	1	1612.43	1471	2.97	0.01376	-
	2	4623.5	3040	5.90	0.01105	-
	3	8770.76	4400	7.99	0.00910	-
N-150-EAP-100	1	1825.27	1487	4.17	0.01544	3379.7
	2	13,437.6	2822	4.43	0.00597	9202.1
	3	10,999.33	4461	3.67	0.00348	17,224.9
N-150-EAP-50	1	2848.79	1476	4.80	0.01474	3896.9
	2	4100.79	2761	5.24	0.00907	10,514.6
	3	11,784.96	4613	9.07	0.00909	17,957.2

**Table 7:** Shock-tube test results using 3 kg ANFO explosives.

	Front	Rear
N-150	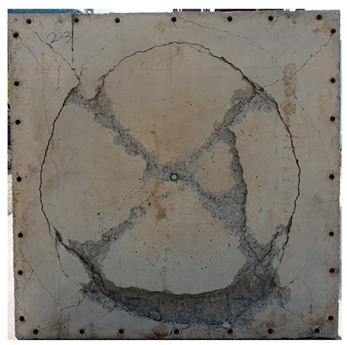	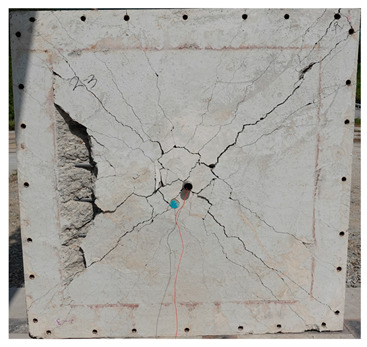
H-150	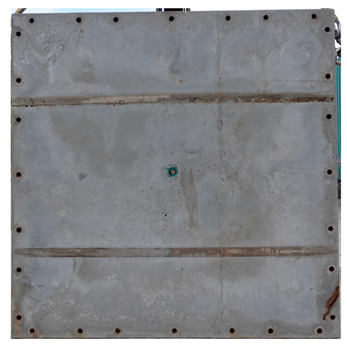	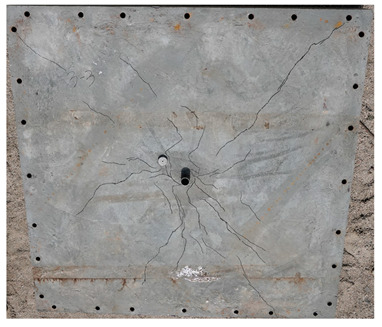
H-100	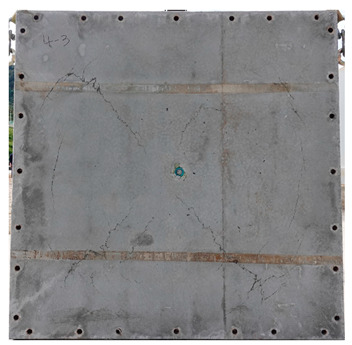	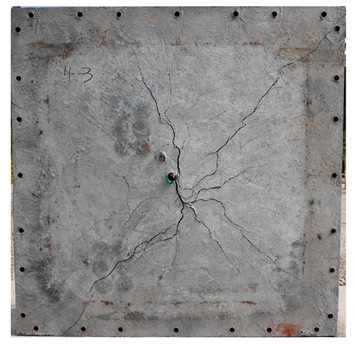
N-150-Po	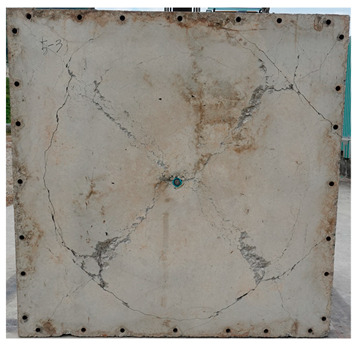	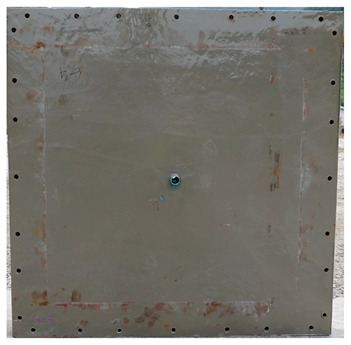
N-150-EAP-100	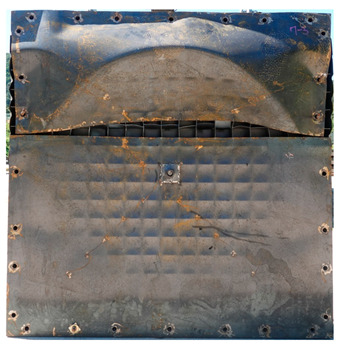	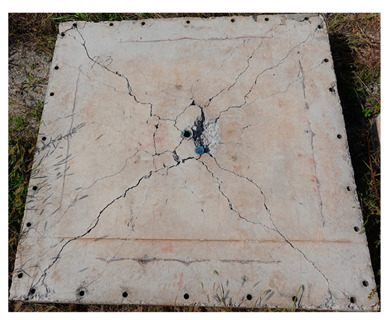
N-150-EAP-50	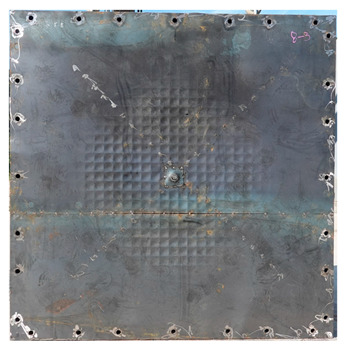	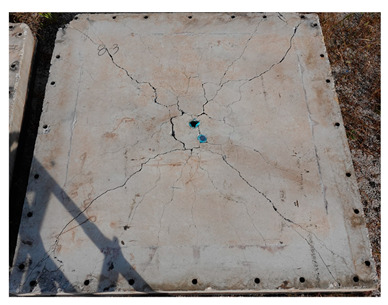

## Data Availability

Not applicable.
